# Clinical and Cost-Effectiveness of Shared Decision Making: Evidence from a Prospective Multicenter Study Evaluating a Hospital-Based Intervention in Germany

**DOI:** 10.1177/0272989X261450971

**Published:** 2026-06-05

**Authors:** Marie Barzen Coors, Fülöp Scheibler, Jens U. Rüffer, Kai Wehkamp, Udo Schneider, Wiebke Schüttig, Friedemann Geiger, Leonie Sundmacher

**Affiliations:** Department of Health Economics, TUM School of Medicine and Health, Technical University of Munich, Munich, BY, Germany; Munich Center for Health Economics and Policy, Munich, BY, Germany; National Competency Center for Shared Decision Making, University Hospital Schleswig-Holstein, Kiel, SH, Germany; TAKEPART Media + Science GmbH, Cologne, NRW, Germany; ISPP Institute for Safety of Patients and Health Professionals, MSH Medical School, Hamburg, HH, Germany; LOHMANN konzept GmbH, Hamburg, HH, Germany; Health Services Management, Techniker Krankenkasse, Hamburg, HH, Germany; Department of Health Economics, TUM School of Medicine and Health, Technical University of Munich, Munich, BY, Germany; Munich Center for Health Economics and Policy, Munich, BY, Germany; National Competency Center for Shared Decision Making, University Hospital Schleswig-Holstein, Kiel, SH, Germany; Department of Paediatrics I, University Hospital Schleswig-Holstein, Kiel, SH, Germany; Department of Psychology, MSH Medical School Hamburg, Hamburg, HH, Germany; Department of Health Economics, TUM School of Medicine and Health, Technical University of Munich, Munich, BY, Germany; Munich Center for Health Economics and Policy, Munich, BY, Germany

**Keywords:** shared decision making, healthcare innovation, health policy, health economics, cost effectiveness

## Abstract

**Objective:**

To evaluate the clinical and cost-effectiveness of SHARE TO CARE (S2C), a complex intervention for hospital-wide, systematic implementation of shared decision making.

**Methods:**

We analyzed clinical effectiveness, health care resource utilization, and implementation costs of S2C from the statutory health insurance perspective using a quasi-experimental difference-in-differences approach with evidence from the Department of Neurology. Clinical outcomes included inpatient hospital admissions, emergency department admissions, and rates of standard and advanced imaging procedures. Implementation costs comprised those related to the conception, development, process integration, ongoing support, and auditing of S2C. Health care utilization data covered inpatient and outpatient care, pharmaceuticals, therapeutic services, assistive devices, and nursing care. We conducted sensitivity analyses to account for uncertainties.

**Findings:**

S2C was associated with a reduction in inpatient hospital admissions, emergency department admissions, and imaging rates in the intervention group. The cost analyses aligned with these findings, showing reduced total costs and health care resource utilization in the intervention group. Although none of the estimates reached the predefined thresholds for statistical significance, the primary analysis yielded weak evidence (*P* < 0.1) of a reduction in emergency department admissions in the intervention group. Overall, savings outweighed the costs of implementing S2C, suggesting cost-effectiveness.

**Conclusions:**

S2C has the potential to reduce emergency department admissions and overall health care costs from the statutory health insurance perspective. Further research should investigate generalizability, the timing of the treatment effect, and potential biases introduced by the COVID-19 pandemic. The demonstrated effects of shared decision making (SDM) have encouraged statutory health insurances in Germany to offer additional reimbursement for clinics certified under the S2C program. The S2C model illustrates how payers and providers can collaborate to facilitate the nationwide implementation of SDM.

**Highlights:**

- The implementation of SHARE TO CARE (S2C) was associated with a statistically nonsignificant reduction in emergency department admissions after 1 y from the statutory health insurance perspective, based on data from the Department of Neurology.
- The cost savings from reduced health care utilization outweighed the implementation costs, and despite not reaching statistical significance, the results support the potential cost-effectiveness of S2C.
- S2C has the potential for nationwide implementation as a systematic form of shared decision making.
- Future research should investigate the generalizability of the results to other health care settings.

## Introduction

Shared decision making (SDM) is a collaborative approach to health care in which clinicians and patients jointly make decisions based on clinical evidence alongside patients’ values and preferences.^1^ With health care systems worldwide seeking to improve the quality and efficiency of care, SDM has gained attention as an approach that can improve clinical effectiveness, quality of care, adherence to treatment, and patient satisfaction.^2–4^ In terms of economic efficiency, large-scale studies conducted in the United States indicate that the costs of implementing SDM programs could be offset by improved appropriateness and necessity of medical care and a reduction in overtreatment.^5,6^

In 2013, Germany established the legal framework for SDM through the enactment of the Patients’ Rights Act (BGB §630). This legislation aims to strengthen patient protection while enhancing patient participation and access to information. However, the systematic and nationwide implementation of SDM is lacking, and patients are still rarely involved in health decision-making processes in a meaningful way.^7–9^ Furthermore, statutory health insurers have not yet established uniform financial incentives to encourage clinicians to engage in SDM more strongly.

The project “Making SDM a Reality” developed a comprehensive approach for implementing SDM on a hospital-wide scale in Germany. Its central intervention was the multicomponent SHARE TO CARE (S2C) program, which consists of 4 modules designed to train physicians, nursing, and other nonphysician medical staff as well as patients in the principles of SDM while facilitating the delivery of patient-centered, evidence-based care.^10^ The implementation of SDM through the S2C program was found to be feasible, sustainable, and effective in terms of patient-perceived outcomes both during the program and beyond.^11–13^ The aim of the present study was to assess the clinical effectiveness and associated costs of the S2C program for 1 y from the statutory health insurance perspective.

## Methods

### Setting and Intervention

We used a quasi-experimental difference-in-differences (DiD) approach to evaluate prospectively collected data on the clinical effectiveness and costs of the S2C program.

S2C was implemented sequentially across 22 hospital departments in the inpatient sector at the University Hospital Schleswig-Holstein (UKSH), Campus Kiel, between 2018 and 2021. Although our original analysis specified that patients from all departments should be included, the COVID-19 pandemic substantially disrupted health care delivery in Germany and introduced significant challenges that affected both clinical operations and study execution. These included shifts in patient volumes, changes in treatment pathways, staffing shortages, and evolving hospital priorities. As a result, we chose to analyze patients who received treatment in a department where implementation was completed ahead of the pandemic-related distortions, that is, the Department of Neurology. As prescribed in the study protocol,^14^ it was the first department at UKSH to implement S2C. Implementation in the department was completed in December 2019, allowing for 1 full quarter of SDM care (January–March 2020). During this period, the department demonstrated full participation in training, stable leadership engagement, and consistent integration of SDM tools into clinical workflows. As such, it provided the most stable and complete context for assessing the program’s impact.

Data were collected during 2 identification periods for the intervention group and the control group. The first identification period (t_0_: July 2017 to June 2018) served as the baseline before the intervention, and the second identification period occurred after the intervention (t_1_: January 2020 to March 2020). Patients were followed for 1 y after being identified in the dataset. Throughout the article, the observation period refers to the 1-y follow-up after the date of patient identification, during which outcomes were measured.

The multicomponent S2C program consists of 4 modules targeting physicians, nurses, and other nonphysician medical staff and patients. These modules are designed to facilitate transdisciplinary decision making based on the “The Six Steps of SDM,” an approach whose individual components have been tested in randomized controlled trials.^10^ Module 1 involves training physicians through independent assessments of videotaped patient consultations, followed by individualized feedback and online courses to strengthen SDM competencies. Module 2 provides patients with evidence-based online patient decision aids to improve their understanding of their health conditions and the available treatment options.^15^ Module 3 trains nurses and other nonphysician medical staff in SDM and their roles in facilitating SDM between doctors and patients. In addition to this basic curriculum for all nurses and other nonphysician medical staff, selected participants attended a 2-d workshop to be trained as decision coaches.^16^ Module 4 aimed to actively engage patients in their consultations with physicians by using the “Ask 3 Questions” approach.^17^ Departments that successfully completed all 4 modules were awarded an S2C certificate.

In total, 92% of all doctors (*n* = 42) in the Department of Neurology at the UKSH Kiel completed the training program. By thus meeting the predefined minimum threshold of 80%, the department was awarded the S2C certificate. In addition, 10 decision aids were developed, covering topics such as the treatment of epilepsy, neuropathic pain, Parkinson’s disease, carotid artery stenosis, and multiple sclerosis. A comprehensive list of the decision aids is available on the S2C Web site (https://share-to-care.de/programm).

Due to the hospital-wide implementation of the program, patients in the control group did not receive their medical care at the UKSH Kiel. Instead, we selected 47 comparable university and teaching hospitals throughout Germany to serve as controls (a list of these hospitals is available upon request). Selection criteria included care structure (university or teaching hospital), geographic location (to minimize potential spillover effects), and hospital size (e.g., number of beds, inpatient and outpatient volumes).

Further details on the study design and program modules have been published elsewhere.^14^ Ethical approval was obtained from the Medical Ethics Committee of the Medical Faculty of Christian-Albrecht University Kiel.

### Data Availability and Study Sample

Techniker Krankenkasse (TK), the largest statutory health insurer in Germany,^18,19^ provided the administrative claims data for this analysis. The pseudonymized data included information on patient sociodemographic characteristics, inpatient and outpatient care, diagnoses, comorbidities, pharmaceutical prescriptions, and costs. For each identification period (t_0_ and t_1_), individual patient-level claims data were available for the 1 y before and the 1 y after patient identification. To ensure data completeness, the study sample included only patients with continuous insurance coverage with TK throughout the observation periods.

We defined the study population using a 3-step procedure based on predetermined inclusion and exclusion criteria. First, we identified the intervention group for both observation periods (t_0_ and t_1_) using a cross-sectional approach. To be eligible for the intervention group, patients had to be adults (≥18 y) hospitalized in the Department of Neurology at the UKSH Kiel. Second, we constructed the respective control group for t_0_ and t_1_ using an exact matching method.^20^ Each intervention patient was matched to up to 5 control patients without replacement. To be eligible for the control group, patients had to be adults hospitalized at the Department of Neurology at one of the control hospitals. Hospitalizations for the intervention and control patients had to occur during the same quarter. To ensure comparability between the study groups while maintaining the integrity of the study design, we selected matching criteria that were time invariant and independent of the intervention assignment.^21^ The demographic criteria comprised predefined age groups and sex. Due to the scope and complexity of the ICD 10-GM catalogue, it was not feasible to use the primary diagnosis for the hospital admission or morbidity measure as a clinical matching criterion, as specified in the original study protocol.^14^ Instead, we used the length of hospital stay for this purpose.

Finally, in the third step, we applied exclusion criteria. Patients with cognitive or psychiatric diagnoses (ICD 10-GM F codes) were excluded because of potential limitations in their ability to actively participate in the decision-making process. In addition, to avoid group-specific distortion of average costs due to high-cost outliers, we excluded patients whose total health care costs were greater than the 99th percentile.

### Economic Evaluation

The aim of this study is 3-fold: first, to evaluate the clinical effectiveness of the S2C program using prespecified outcome parameters^14^; second, to calculate the costs associated with the S2C intervention and patients’ utilization of health care resources; and third, to determine the cost-effectiveness of the new model of care compared with standard care within 1 y of its implementation, from the statutory health insurance perspective. The study design adhered to the Consolidated Health Economic Evaluation Reporting Standards.^22^

#### Effectiveness

We investigated 4 utilization patterns to assess the clinical effectiveness of S2C: inpatient hospital admissions, emergency department admissions, and standard and advanced imaging rates.

Consistent with prior research, we anticipated that the provision of evidence-based information and patient involvement in decision making would enhance health literacy,^4^ improve patient safety,^23^ and increase the appropriateness and necessity of medical care. These improvements are expected to reduce overtreatment and promote more efficient resource utilization.^5,6^ Accordingly, we anticipated reductions in emergency department admissions, hospital admissions, and imaging rates.

We identified inpatient hospital admissions based on hospital case records in the administrative claims data. Inpatient emergency admissions were distinguished using the documented reason for admission. Standard inpatient and outpatient imaging rates comprised X-rays and ultrasound examinations, whereas advanced imaging rates comprised computed tomography, magnetic resonance imaging, diagnostic positron emission tomography, and nuclear medicine diagnostic procedures. We identified all imaging procedures using the German procedure classification code (OPS: Operationen- und Prozedurenschlüssel) and the German Uniform Value Scale (EBM: einheitlicher Bemessungsmaßstab).

#### Costs

To estimate total costs from the statutory health insurance perspective, we considered S2C implementation costs and health care resource utilization. Based on the anticipated effects, we expected that reductions in the clinical effectiveness measures would result in a decrease in overall health care expenditures. Furthermore, we anticipated that reductions in health care resource utilization would outweigh the costs of implementing S2C.

The measured costs of the S2C program were the expenses related to the conception, development, process integration, rollout (including staff training), ongoing support, and auditing of all 4 modules. Training costs included compensated physician training time, calculated at salary-equivalent rates for dedicated training sessions. Training of nurses and other nonphysician staff was integrated into regular team meetings and working hours; no additional personnel costs were assigned for these groups. We refer to these activities collectively as “implementation.” Costs related to administering and conducting the evaluation study were excluded. We calculated average implementation costs per patient using a lump-sum approach at the hospital level, dividing the total implementation costs by the number of treated patients during the t_1_, as documented in trial records and funding documentation.

To ascertain the costs associated with the utilization of health care resources, we multiplied the unit costs by the observed utilization rates documented in the administrative claims data. These data included costs for inpatient and outpatient care, pharmaceuticals, assistive devices and rehabilitative therapies, and nursing care. We derived inpatient care costs from the German diagnosis-related group (DRG) classification system and outpatient care costs from the documented EBM lump-sum compensation and additional material costs, excluding those for dialysis. Pharmaceutical costs reflected the prices paid by TK.

We calculated total costs as the sum of the S2C implementation costs and the costs of health care resource utilization. For patients who died during the observation period, we prorated costs up to the date of death. To account for inflation, we adjusted all costs incurred between 2017 and 2021 using the German Harmonised Index of Consumer Prices, with 2021 as the reference year. Owing to the 1-y observation period, discounting of costs and effectiveness measures was not applicable.

Further details on the definitions, operationalization, measurement, and valuation of the clinical outcome and cost parameters are provided in Table A1 in Supplement A.

#### Statistical analysis

We employed a quasi-experimental evaluation method using a DiD approach. Using a repeated cross-sectional setup, we compared clinical and cost parameters between the intervention group and control group before and after the intervention. A simplified representation of the DiD model is shown in Supplement A.

The validity of the DiD approach relies on several key assumptions commonly used in causal inference studies, including the parallel trends assumption, the stable unit treatment value assumption (SUTVA), the exogeneity of conditioning variables, and the “common shocks” assumption.^24,25^ The parallel trends assumption posits that, in the absence of the intervention, the difference in outcomes between the intervention group and control group would have remained constant over time. This assumption allows the observed changes in the control group to serve as a valid counterfactual for the intervention group.^25^ In turn, SUTVA requires that the implementation of S2C in the intervention group does not interfere with outcomes in the control group and that the treatment is consistently applied in the intervention group. The assumption that the conditioning variables are exogenous assumes that the covariates included in the analysis are not influenced by the intervention and remain independent of treatment assignment (e.g., patient demographic characteristics or pretreatment health care costs must not be systematically altered by the intervention or its implementation process). Lastly, the “common shocks” assumption states that the intervention group and control group must be exposed to the same external influences (e.g., health care trends, policy changes, or macroeconomic shocks) over the study period. This ensures that any observed differences in outcomes can be attributed to the intervention rather than unrelated external factors.

To evaluate the validity of these assumptions, we compared the observable preperiod characteristics of the matched study groups before and after the intervention to identify potential sociodemographic and clinical confounders that could influence treatment assignment or outcome trends. Subsequently, characteristics that were unit specific and time variant were included as covariates in multivariate regression models.^25^ The sociodemographic characteristics included in the analyses were patient age, sex, mortality status (alive or deceased), and retirement status. Clinical characteristics comprised the long-term care needs level (measured on a 6-point scale of 0 to 5), preperiod hospitalization days, preperiod health care costs, and the preperiod Elixhauser Comorbidity Index.^26^

To empirically measure the impact of S2C, we specified the following DiD regression model:

(1) $$\begin{array}{c} Y_{\mathrm{igt}}=\beta_{0}+\beta_{1}\mathrm{Grou}p_{g}+\beta_{2}\mathrm{Pos}t_{t}+\delta \left(\mathrm{Grou}p_{g}*\mathrm{Pos}t_{t}\right)\\ \;\;\;+\beta_{3}X\prime_{\mathrm{igt}}+\varepsilon_{\mathrm{igt}}\left(1\right) \end{array}$$

where $Y_{\mathrm{igt}}$ represents the outcome or cost parameter for individual i in group g during treatment period t . $\mathrm{Grou}p_{g}$ is a binary variable indicating whether the individual belongs to the intervention group, and $\mathrm{Pos}t_{t}$ is a binary variable indicating whether period t is after the implementation of S2C. The coefficient ${\hat{\beta }}_{1}$ accounts for baseline differences between the study groups, and ${\hat{\beta }}_{2}$ represents the time trend. The main coefficient of interest is the DiD estimate denoted as $\hat{\delta }$ , which represents the average treatment effect on the treated. Vector $X_{\mathrm{igt}}^{\prime }$ comprises the identified covariates, and $\varepsilon_{\mathrm{igt}}$ is the error term. We applied robust standard errors to account for potential heteroskedasticity in the data.^27^

The functional form of the linear regression model was tailored to the respective distribution of the outcome and cost parameters. For the count data, we employed a generalized linear model (GLM) with a Poisson distribution. For the cost data, to account for its nonnegative, right-skewed distribution, we applied a gamma-distributed GLM. We determined the model specifications for the main analysis using the Akaike information criterion and Bayesian information criterion as relative measures of goodness-of-fit criteria, and we explored alternative model specifications as part of the sensitivity analyses.^28^ In addition, we assessed the goodness of fit of the link function using the Pregibon^29^ link test.

For statistically significant DiD estimates, we performed a cost-effectiveness analysis by dividing the incremental costs by the incremental effects. The resulting incremental cost-effectiveness ratios represent the incremental costs per avoided hospitalization, emergency department admission, or imaging procedure.

#### Sensitivity analysis

We applied a multidimensional approach to investigate the sensitivity and robustness of our results.

First, we examined the credibility of the counterfactual assumption by observing the preintervention outcome trends by group over time.^30^ Figure A2 in Supplement A provides a graphical representation of the mean outcomes for patients during t_0_. In the absence of the S2C intervention, we observed parallel trends for inpatient hospital admissions, emergency department admissions, and standard imaging rates, as well as similar trends for advanced imaging rates and total healthcare costs. These observations suggest that the previously described matching and identification strategy produced comparable study groups with consistent outcome trends as would be expected in the absence of treatment.

Second, we evaluated the robustness of our analysis with respect to the statistical specifications of the DiD model. In lieu of assuming a Poisson distribution for the clinical outcome variables, we applied a negative binomial GLM to account for potential overdispersion.^31^ Furthermore, the specification of the main model assumed independent errors across observations, which could lead to downward bias and overrejection of the null hypothesis in clustered designs.^30,32^ Because cluster-robust standard errors are known to perform poorly in settings with only 1 treated unit,^33^ we investigated the impact of intracluster correlation on inference using bias-adjusted cluster-robust standard errors.^34^

Third, we adapted the DiD model to account for clinically relevant specifications. Although the Elixhauser Comorbidity Index is a validated and widely used measure for depicting the burden of disease, the results may be sensitive to the instrument selected. We thus applied the Charlson Comorbidity Index (CCI) as an alternative measure.^35^ Furthermore, the postintervention observation period coincided with the emergence of the COVID-19 pandemic in Germany. Because the severity of the pandemic and associated restriction measures varied across Germany, we attempted to map the region-specific dynamics and their impact on the provision of medical care by controlling for COVID-19 diagnoses at the patient level.

We interpreted our findings based on their clinical relevance and statistical significance, applying a 2-sided significance level of 5%. Results significant at the 10% level were reported as weak evidence. All analyses were conducted using Stata 16.1 and R version 4.3.2.

## Results

### Study Population

**Figure 1:**
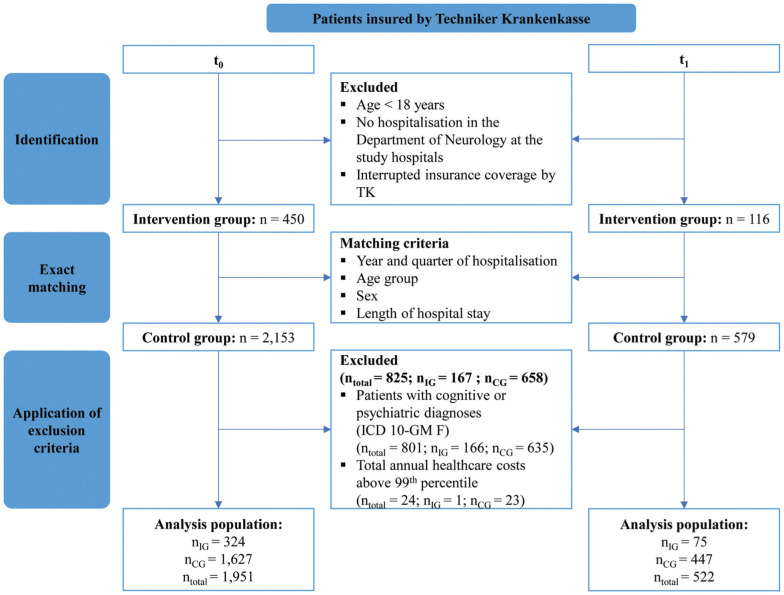
Study population flow chart.

The identification and matching strategy yielded a total of 2,474 analyzable patients, of whom 1,951 were observed during t_0_ and 522 during t_1_. The ratio of intervention to control patients remained approximately 1 to 5 after we applied the exclusion criteria. Figure 1 provides a graphical summary of the study population.

Table 1 summarizes the characteristics of the study population. The average patient age was 61.5 y, with approximately half of the patients being female and retired. Between 20% and 32% of patients had a long-term care needs level of at least 1 on a scale of 0 to 5. The mortality rate 12 mo after patient identification ranged from 3% to 8%. Patients observed during t_0_ spent, on average, fewer days in the hospital, resulting in lower total health care costs compared with patients observed during t_1_.

**Table 1:** Characteristics of the Study Population

Parameter	t_0_		t_1_		
	Intervention^a^	Control^a^	Intervention^a^	Control^a^	DiD^b^
Sociodemographic characteristics					
Age^c^	61.95 (18.18)	62.29 (17.75)	60.08 (19.20)	62.04 (19.12)	−1.6 [−6.8, 3.5]
Sex^d^ (0 = female, 1 = male)	0.54 (0.50)	0.52 (0.50)	0.49 (0.50)	0.57 (0.49)	−0.38 [−0.93, 0.16]
Retired^d^ (0 = no, 1 = yes)	0.52 (0.50)	0.53 (0.50)	0.48 (0.50)	0.50 (0.50)	−0.03 [−0.58, 0.51]
Long-term care needs^d^ (0 = no, 1 = yes)	0.22 (0.41)	0.26 (0.44)	0.20 (0.40)	0.32 (0.47)	−0.39 [−1.1, 0.27]
Mortality rate^d^	0.03 (0.18)	0.07 (0.26)	0.08 (0.27)	0.08 (0.27)	0.88 [−0.22, 2.0]
Pretrial clinical characteristics					
Length of hospital stay (days)^e^	6.37 (29.47)	5.79 (17.00)	11.72 (47.93)	10.46 (27.83)	0.02 [−1.1, 1.1]
Elixhauser Comorbidity Index^e^	2.98 (2.67)	2.87 (2.57)	2.85 (2.38)	3.42 (2.92)	−0.22* [−0.45, 0.01]
Healthc are costs in €^f^	7,542.96 (15,310.88)	7,830.06 (14,395.86)	11,999.26 (30,811.93)	13,145.14 (31,156.95)	−0.05 [−0.72, 0.61]
*N*	324	1,627	75	447	2,473

DiD, difference-in-differences estimate.

a Mean (SD).

b 95% confidence interval in square brackets.

c Linear regression model.

d Logistic regression model.

e Poisson-distributed generalized linear model with log-link.

f Gamma-distributed generalized linear model with log-link.

* *P* < 0.1.

We did not observe differences between the intervention group and control group over time in terms of age, sex, retirement status, long-term care needs level, mortality, pretrial length of hospital stay, or pretrial health care costs. This lack of differences was expected due to the identification and matching procedures. However, we observed tendencies toward unit-specific and time-varying differences in the pretrial Elixhauser Comorbidity Index. Because the Elixhauser Comorbidity Index is expected to be associated with the outcome parameters, this represented a potential threat to the counterfactual assumption. To address this, we incorporated the patient-level pretrial Elixhauser Comorbidity Index as a covariable (represented by **$X_{\mathrm{igt}}^{\prime }$** in (1)) in the DiD model.

### Effectiveness Analysis

Table 2 presents the average outcome parameters, as well as the primary estimates from the regression models. Over the 12 mo following the identifying hospitalization, patients were hospitalized an average of 0.53 to 0.75 times, with approximately half of these admissions classified as emergencies. Moreover, patients underwent an average of nearly 2 standard imaging procedures and 1.3 advanced imaging procedures during the observation period.

**Table 2:** Hospital Admissions, Emergency Department Admissions, and Standard and Advanced Imaging, by Observation Period and Group

Parameter	t_0_		t_1_		
	Intervention^a^	Control^a^	Intervention^a^	Control^a^	DiD^b,c^
Inpatient hospital admissions					
Total	0.73 (1.30)	0.75 (1.31)	0.53 (0.89)	0.66 (1.18)	−0.09 [−0.33, 0.15]
Emergency department admissions					
Total	0.44 (0.95)	0.33 (0.79)	0.24 (0.49)	0.30 (0.76)	−0.16* [−0.31, 0.00]
Standard imaging					
Total	1.98 (3.25)	1.94 (2.55)	1.85 (2.69)	1.98 (2.82)	−0.10 [−0.83, 0.63]
Inpatient care	0.20 (1.75)	0.13 (0.65)	0.03 (0.16)	0.14 (0.69)	/
Outpatient care	1.78 (2.68)	1.81 (2.44)	1.83 (2.67)	1.84 (2.72)	/
Advanced imaging					
Total	1.34 (2.70)	1.48 (2.85)	1.24 (2.82)	1.47 (2.63)	−0.16 [−0.80, 0.49]
Inpatient care	0.65 (1.75)	0.63 (1.97)	0.61 (1.92)	0.56 (1.67)	/
Outpatient care	0.66 (1.90)	0.84 (1.93)	0.57 (1.29)	0.91 (1.89)	/
*N*	324	1,627	75	447	2,473

DiD, difference-in-differences estimate.

a Mean (SD).

b 95% confidence interval in square brackets.

c Poisson-distributed generalized linear model with identity link, robust standard errors, and adjusted for the pretrial Elixhauser Comorbidity Index.

* *P* < 0.1.

All observed DiD estimates were negative, indicating a potential reduction in the examined outcomes. None of these estimates reached statistical significance at the predefined significance level of 5%. However, we observed weak evidence of a reduction (−0.16, *P* = 0.05) in the number of emergency department admissions. Detailed regression results, including estimates for the covariates, are provided in Table B1 of Supplement B.

### Cost Analysis

**Figure 2:**
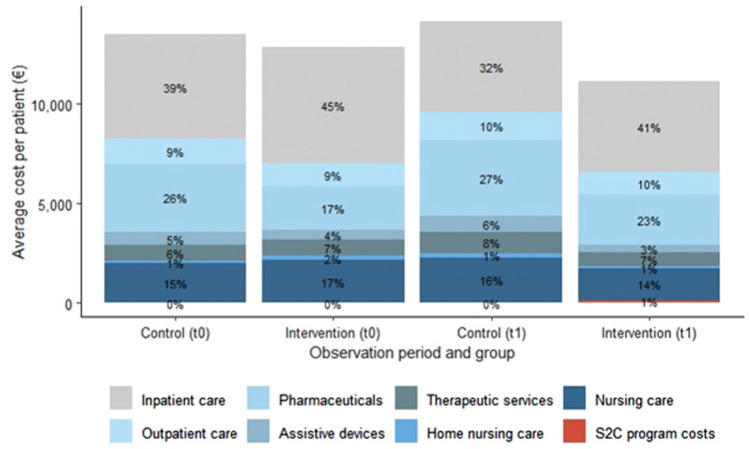
Average total cost per patient, by observation period and group.

Figure 2 depicts the average total cost per patient. We observed the largest proportion of total costs in the inpatient care sector, followed by nursing care, outpatient care, and pharmaceuticals. Costs associated with therapeutic services, assistive devices, and home nursing care accounted for less than 10% of the total costs. The average cost amounts for each of the cost categories are presented in Table B2 of Supplement B.

Table 3 summarizes the total average costs, average health care resource utilization costs, and average S2C implementation costs per group and observation period, accompanied by the respective DiD estimates. The mean cost of implementing S2C per treated patient was €122, with more than 70% attributable to the development and maintenance of the online patient decision aids (Figure B1). The annual costs associated with the utilization of health care resources ranged from an average of €12,846.86 to €14,171.88, depending on the study group and observation period. For intervention group patients in t_1_, we calculated total costs by adding the costs of implementing the S2C program to health care resource utilization costs. Consistent with the findings of the clinical effectiveness analysis, we observed negative DiD estimates for total costs and the costs of health care resource utilization, indicating the potential of the S2C program to lead to savings from the statutory health insurance perspective. However, none of these results achieved statistical significance. The detailed regression results are provided in Supplement B (Table B3).

**Table 3:** Costs of Health Care Utilization, by Observation Period and Group

Parameter	t_0_		t_1_		
	Intervention^a^	Control^a^	Intervention^a^	Control^a^	DiD^b,c^
Total costs in €					
Total	12,846.86 (19,370.70)	13,492.43 (22,676.81)	11,134.15 (19,678.37)	14,171.88 (21,577.33)	−2,395 [−5,508, 717]
Health care resource utilization in €					
Total	12,846.86 (19,370.70)	13,492.43 (22,676.81)	11,012.15 (19,678.37)	14,171.88 (21,577.33)	−2,538 [−5,643, 567]
S2C implementation costs in €					
Total	0.00	0.00	122.00	0.00	/
*N*	324	1,627	75	447	2,473

DiD, difference-in-differences estimate; S2C, SHARE TO CARE.

a Mean (SD).

b 95% confidence interval in square brackets.

c Gamma-distributed generalized linear model with identity link, robust standard errors, and adjusted for the pretrial Elixhauser Comorbidity Index.

* *P* < 0.1.

### Sensitivity Analysis

**Figure 3:**
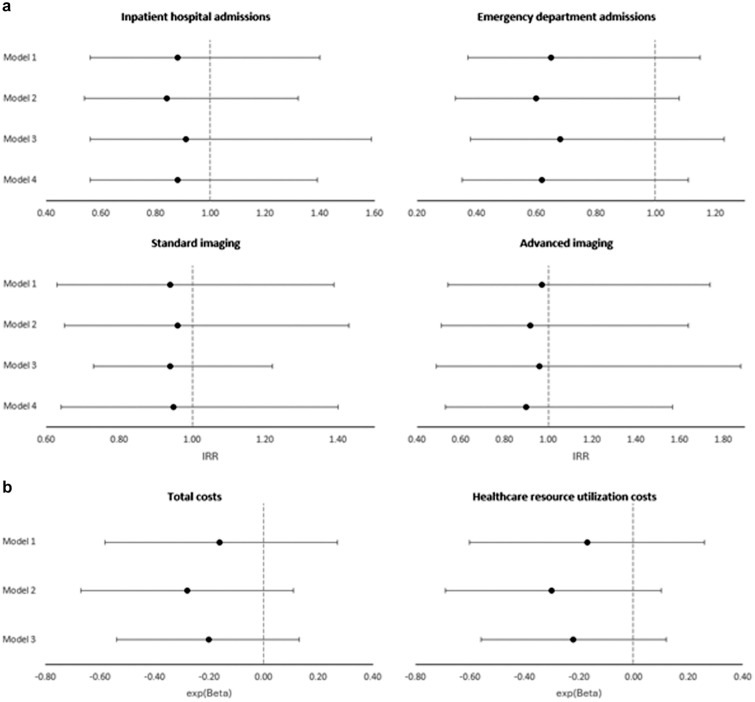
(a) Main results of the sensitivity analyses: (a) outcome parameters and (b) costs.

Figure 3a and b present the DiD estimates for each sensitivity analysis model for each parameter. Further details of the regression models are available in Tables B4 to B9 of Supplement B.

For the clinical outcome parameters, we used 4 sensitivity models, each of which deviated from the main model: Model 1 used the CCI as an alternative measure of comorbidity. Model 2 included an indicator for a COVID-19 diagnosis as an additional covariate. Model 3 used bias-adjusted cluster-robust standard errors to account for potential within-cluster correlation. Model 4 assumed a negative binomial GLM with a log-link function rather than the Poisson distribution used in the main model. The results of the main model were robust to each of the sensitivity analyses; however, only model 2 was able to confirm the weak significant reduction in emergency department admissions.

For the cost parameters, models 1 to 3 were consistent with the sensitivity model specifications applied to the clinical outcomes, whereas model 4 was not applicable to cost parameters. Again, the estimates from the main model were robust to these alternative specifications, with findings indicating a negative but nonsignificant DiD estimate.

### Cost-Effectiveness Analysis

The objective of the cost-effectiveness analysis was to compare incremental costs with incremental benefits. Although we identified a weakly significant reduction in emergency department admissions, this finding was not robust to our sensitivity analyses. The results of the cost analysis suggested a reduction in costs from the statutory health insurance perspective, but no statistically significant effects were observed. As outlined in the “Methods” section, no additional cost-effectiveness analyses were conducted due to the nonsignificant results.

## Discussion

SDM is a recommended method for delivering evidence-based, patient-centered care. Numerous studies have demonstrated the positive effects of SDM on a wide range of outcomes, as summarized in regularly updated systematic reviews.^2,36–38^ In addition, many countries, including Germany, have enacted legislation to support the implementation of SDM. The World Health Organization^39^ also recommends SDM as a strategy for improving patient safety. Despite these developments, SDM has not yet been systematically and fully integrated into the standard of care in Germany.

The German Innovation Fund project “Making SDM a Reality” successfully implemented the multicomponent S2C program at the UKSH, Campus Kiel, in Germany.^11^ This project introduced an evidence-based, hospital-wide approach to systematically integrate SDM into routine clinical practice through targeted interventions for physicians, nurses, and other nonphysician medical staff and patients. Using a DiD approach, we analyzed the clinical effectiveness and costs associated with the implementation of S2C. The results suggest reductions in inpatient hospital admissions, emergency department admissions, and imaging rates in the intervention group compared with the control group. The cost analysis supported these findings, indicating reduced total costs and health care resource utilization in the intervention group. Although these reductions were consistent with the anticipated effects, none of the estimates achieved the predefined threshold for statistical significance. However, weak evidence (*P* < 0.1) was observed for a reduction in emergency department admissions in the primary analysis. Beyond statistical significance, such reductions could have clinically and economically meaningful implications, potentially reducing patient burden and costly emergency care. Furthermore, the savings generated by the program outweighed its implementation costs, suggesting its cost-effectiveness.

Our findings contribute to the growing body of research on the costs and clinical effectiveness of SDM. In 2013, Veroff et al.^6^ published the results of a 12-mo randomized investigation demonstrating that improved support for SDM led to a reduction in overall medical costs, with savings exceeding the costs of implementation. Similarly, a recent publication by Brown et al.^40^ confirmed that SDM lowers annual total health care expenditures. Consistent with our findings, they reported a reduction in emergency department admissions as a possible contributing factor.

One potential facilitator for the observed reduction in total costs and emergency department admissions is the increased level of patient health literacy following the implementation of S2C.^41^ Improved health literacy may reduce the risk of exacerbations, enabling patients to navigate the health care system more effectively and use health care resources more efficiently.^42^ Furthermore, the observed reduction in total health care costs may reflect more efficient resource utilization and a decrease in unwarranted services. In Germany, the pervasive overuse of diagnostic imaging, emergency department visits, and hospital admissions—often in scenarios where outpatient management would have been adequate—has been well documented.^43–48^ One structural driver of such overtreatment in the inpatient setting is the DRG payment system, which incentivizes procedural volume and hospital admissions over care appropriateness.^49^ Accordingly, the reductions observed in our study are consistent with the hypothesized mechanism that SDM can mitigate unnecessary utilization in a health care system in which financial incentives and sectoral structures favor overuse.

The raw results of this study were reported to the German Innovation Fund, and these alongside those of prior evaluations of S2C^11,12^ led them to recommend S2C as a national standard of care.^50^ Furthermore, the demonstrated effects of SDM have encouraged statutory health insurance companies in Germany to offer additional reimbursement for clinics certified under the S2C program. Concurrently, an increasing number of German hospitals have adopted the program. Due to economies of scale and scope, and by building on the methods and materials developed in Kiel, the implementation costs per patient could be considerably lower in broader, routine settings than those measured in this study. Overall, the Kiel model illustrates how payers and providers can collaborate to facilitate the implementation of SDM on a nationwide basis.^23^

### Strengths and Limitations

The strengths of this study include the relevance of the analyzed outcome and cost parameters, the robust quasi-experimental study design that facilitates causal inference, and the evaluation of a program addressing the widely recognized importance of SDM in modern health care.

The quasi-experimental study design benefited from the combined use of exact matching and the DiD approach. Although a randomized controlled trial would have been the preferred approach to investigate the causal effects of the S2C program, this was not feasible due to its hospital-wide implementation and the administrative burden for potential control clinics. As a consequence, the intervention hospital was not randomly selected but volunteered to participate. Although this may introduce some degree of selection bias, we addressed this by selecting 47 structurally similar control hospitals to enhance comparability. Furthermore, the combination of exact matching and the DiD approach allowed us to draw on the strength of both methods, yielding robust inferences, reducing confounding, and improving accuracy.^32,51–53^ To avoid regression to the mean bias when applying matching strategies on preperiod variables as part of our DiD approach, we adhered to the recommendations of Daw and Hatfield^21^ when selecting the matching parameters.

The available dataset provided detailed information on health care resource utilization from the statutory health insurance perspective. However, administrative claims data are not specifically collected for research purposes and are thus susceptible to bias due to variations in coding practices or documentation quality. For example, although we used the recommended procedure to identify inpatient emergency department admissions based on the reason for admission,^54^ the lack of a standardized protocol or clinical practice standards may limit comparability. Furthermore, the dataset constrained our sample to patients insured by TK. Although TK has a substantial market share,^18^ patient characteristics may vary between statutory health insurers, limiting the generalizability of the results. In addition, the number of observed patients may have limited the statistical power of the analysis.

The reported implementation costs reflect the direct expenses that were actually paid as part of the project. However, due to data limitations, we were not able to capture further opportunity costs, particularly those associated with the time of nurses and other nonphysician staff (e.g., time spent in training during regular working hours and in the 2-d decision coach workshop). This time was taken from other tasks and was thus a cost of the intervention. As a result, the reported implementation costs are underestimated.

The COVID-19 pandemic had a substantial impact on the implementation timeline of the S2C program and, thus, the feasibility of the present study. Anticipating substantial distortions resulting from altered health care provision processes and capacity constraints, we shortened the identification period t_1_ to 1 quarter instead of 1 y. As a result, the following parts of the planned analysis in the study protocol could not be conducted:

1. One objective was to assess the impact of S2C on procedures performed for internationally comparable preference-sensitive conditions (PSCs). PSCs involve treatment options that require patients to weigh the tradeoffs between quality of life and survival time,^55^ making patients with PSCs especially suitable for SDM. Although patients with neurologic disorders frequently face preference-sensitive decisions, none of the cases in our sample were consistent with the predefined PSCs in the study protocol, and thus, this analysis could not be conducted.
2. We aimed to analyze the potential heterogeneity of the effect mechanisms of the S2C program on several subgroups. However, the relatively small sample size during the second observation period precluded further stratification of the data.
3. We planned to investigate the timing of the average treatment effect on the treated by dividing the 1-y observation period into quarters. Once again, the restricted sample size during t_1_ prevented this analysis.

In addition to these practical considerations, the COVID-19 pandemic likely affected the predefined outcome parameters. Despite our efforts to account for the regional dynamics of the pandemic by controlling for COVID-19 diagnoses in one of our sensitivity analyses, the observed results may still be influenced by the complex and heterogeneous impact of COVID-19 on care provision and patient behavior.

Although the study has limitations, the project was notable for the scope of the implemented measures and the human and financial resources invested in their realization. To generate insights that are useful and transferable for health care decision makers, future implementation initiatives, and the scientific community, we conducted those components of the evaluation that remained feasible and could meaningfully contribute to the evidence base. Furthermore, the applied methodological approach is transferable to other clinical departments and may serve as a proof of concept for examining the generalizability of the findings.

## Conclusions

This study provides evidence of the clinical and cost-effectiveness of implementing a multicomponent, hospital-wide SDM program. The results suggest that successfully integrating S2C into the standard of care across Germany could reduce emergency department admissions and overall health care costs from the statutory health insurance perspective. However, the findings were not robust to our various sensitivity analyses. Thus, further research is necessary to address the limitations of the study, particularly the generalizability of the results to other clinical departments, the timing of the treatment effect, and potential biases introduced by the COVID-19 pandemic.

### Implications

The demonstrated effects of SDM have encouraged statutory health insurances in Germany to offer additional reimbursement for clinics certified under the S2C program. The S2C model illustrates how payers and providers can collaborate to facilitate the implementation of SDM on a nationwide basis.

## Supplemental Material

sj-docx-1-mdm-10.1177_0272989X261450971 – Supplemental material for Clinical and Cost-Effectiveness of Shared Decision Making: Evidence from a Prospective Multicenter Study Evaluating a Hospital-Based Intervention in Germany Supplemental material, sj-docx-1-mdm-10.1177_0272989X261450971 for Clinical and Cost-Effectiveness of Shared Decision Making: Evidence from a Prospective Multicenter Study Evaluating a Hospital-Based Intervention in Germany by Marie Barzen Coors, Fülöp Scheibler, Jens U. Rüffer, Kai Wehkamp, Udo Schneider, Wiebke Schüttig, Friedemann Geiger and Leonie Sundmacher in Medical Decision Making
