# Supplementary material for: Clinical and Cost-Effectiveness of Shared Decision Making: Evidence from a Prospective Multicenter Study Evaluating a Hospital-Based Intervention in Germany
Source: Med Decis Making. 2026 Jun 5;46(6):795–809. doi: 10.1177/0272989X261450971 (PMC13346598; doi:10.1177/0272989X261450971)
Supplement: sj-docx-1-mdm-10.1177_0272989X261450971 – Supplemental material for Clinical and Cost-Effectiveness of Shared Decision Making: Evidence from a Prospective Multicenter Study Evaluating a Hospital-Based Intervention in Germany [file sj-docx-1-mdm-10.1177_0272989X261450971.docx]

**Supplement A**

Table A1: Technical operationalisation of clinical outcomes and cost variables based on routine data

| Variable | Definition and operationalisation |
| --- | --- |
| **Clinical outcomes** | |
| Inpatient admissions | Number of documented hospital cases per patient, excluding pre-stationary care and intra-hospital or inter-institution transfers  *Operationalisation:* unique case ID |
| Emergency department admissions | Number of inpatient emergency admissions  *Operationalisation:* documented reason for admission according to §301 agreement, Annex 2, key 1 & 5, document: 15th update of the §301 agreement, last change: 12.10.2018 |
| Standard imaging | Number of outpatient and inpatient X-ray and ultrasound examinations (incl. imaging of the vascular system)  *Operationalisation:* OPS and EBM coding   - Inpatient standard imaging: ultrasound examination, projection radiography, imaging of the vascular system - Outpatient standard imaging: ultrasound diagnostics, diagnostic radiology |
| Advanced imaging | Number of outpatient and inpatient computer tomography (CT), magnetic resonance tomography (MRT), diagnostic positron emission tomography (PET), nuclear medicine diagnostic procedures  *Operationalisation:* OPS and EBM coding   - Inpatient advanced imaging: computed tomography, nuclear medicine diagnostic procedures, magnetic resonance imaging, other imaging procedures - Outpatient advanced imaging: computed tomography (supplements excluded), magnetic resonance imaging, non-vascular interventional procedures, Osteodensitometry (bone densitometry), diagnostic positron emission tomography (PET) - Imaging during early detection examinations, maternity care, contraception and abortion were excluded |
| **Costs variables** | |
| Inpatient care costs | *Measurement:* Reimbursements for inpatient hospital services  *Valuation:* services billed via DRG |
| Outpatient care costs | *Measurement:* Reimbursements for outpatient care costs  *Valuation:* services billed via the EBM number shown and the corresponding point value, material costs, dialysis costs |
| Pharmaceuticals | *Measurement*: Reimbursements for medical prescriptions  *Valuation:* documented pharmaceutical central number, multiplication factor, net unit price paid |
| Other care services | |
| Therapeutic services | *Measurement*: Remuneration for therapeutic services  *Valuation:* documented therapeutic product item number (POSNR), number of billing items, net price paid at POSNR level |
| Assistive devices | *Measurement*: Reimbursements for assistive devices  *Valuation:* documented billing item number, number of billing items, net price paid |
| Home nursing care | *Measurement*: Remuneration for home nursing care  *Valuation:* documented billing item number, number of billing items, net price paid |
| Nursing care | *Measurement*: Reimbursements for nursing care services  *Valuation:* documented type of service, net price paid |
| S2C implementation costs | |
| Module 1: Training of physicians | *Measurement*:  Costs related to the conception, development, implementation (including time in training) and auditing of the three-phase training of physicians:   - Phase 1: online training introducing SDM and its six steps - Phase 2: individual coaching based on video recordings of the physician’s own consultations - Phase 3: additional video coaching to reinforce and expand skills   *Valuation:* funding documentation including personnel costs for the conception, development and implementation of the module; compensation records for personnel costs (salary equivalent) of the trained physicians |
| Module 2: Online patient decision aids | *Measurement:*  Costs related to the conception, development, implementation, ongoing support and auditing of the online decision aids. The process was applied to the most common preference-sensitive decisions in each medical specialty and included:   - Patient-friendly texts and graphics - Explanatory video clips by physicians from the treating hospital - Video testimonials from patients sharing their personal experiences and preferences - A value clarification tool   *Valuation:* invoices of the external service provider responsible for the conception, development and implementation of the patient decision aids (including personnel and material costs) |
| Module 3: Training of nurses and other non-physician medical staff | *Measurement:*  Costs related to the conception, development, implementation and auditing of the training of nurses and other non-physician medical staff:   - Video clip during regularly scheduled team meetings including information on how to apply SDM in nursing practice and how to support SDM processes between physicians and patients   Costs related to the conception, development, implementation and auditing of the training of nurses and other non-physician medical staff as decision coaches:   - Two-day workshop to provide information on how to guide patients in using decision aids and help anchor the use of decision aids structurally within the patient pathway   *Valuation:* funding documentation including personnel costs (salary equivalent) for the conception, development and implementation of the module; no additional personnel costs were assigned for these staff groups as part of the project |
| Module 4: Patient engagement | *Measurement:*  Costs related to the conception, development, implementation and auditing of a hospital-wide campaign to proactively engage patients in the decision-making process:   - Printing and circulation of posters, flyers, video clip on waiting room screens   *Valuation:* funding documentation including personnel costs for the conception, development and implementation of the module as well as material costs |

S2C: SHARE TO CARE


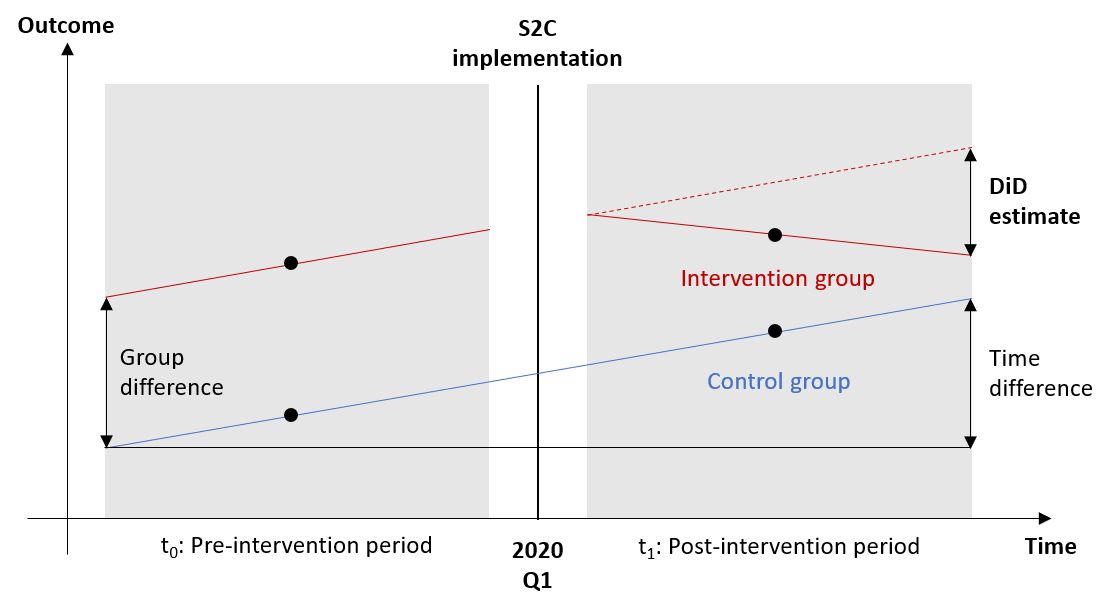


Figure A1: Graphical illustration of the DiD identification strategy


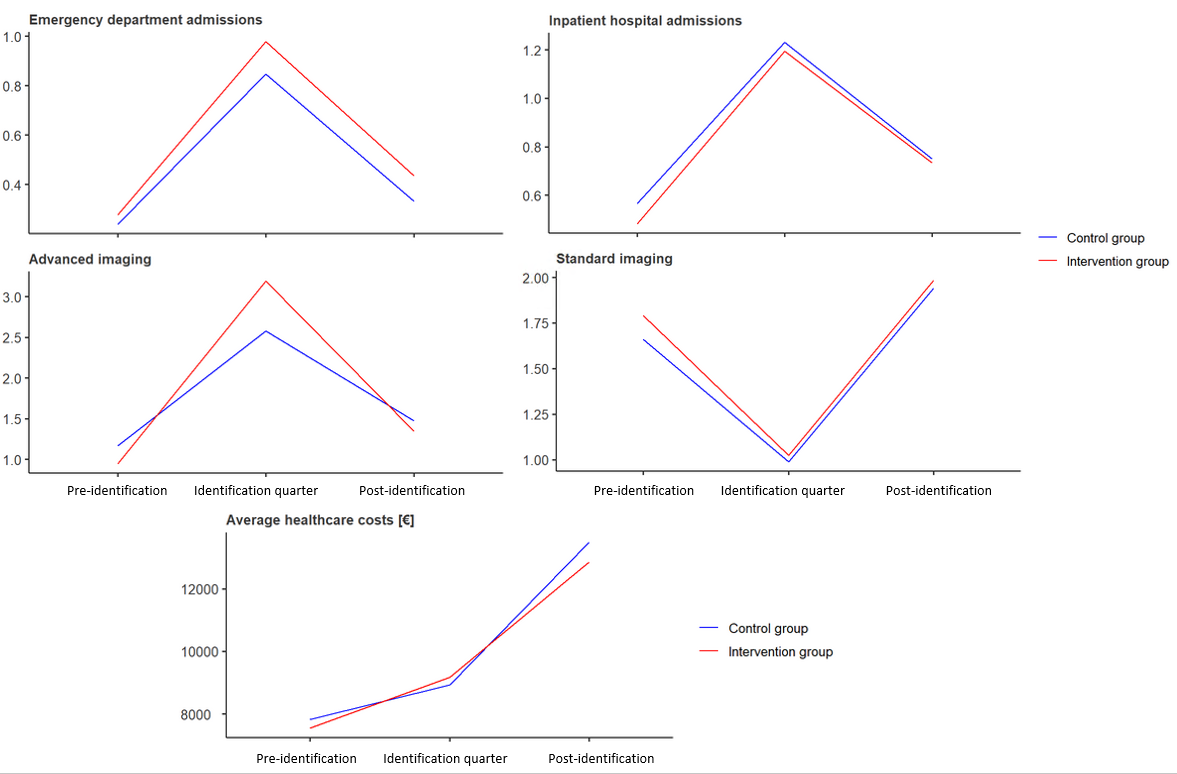


Figure A2: Graphical representation of the mean outcomes of patients observed during the pre-intervention period (t_0_)

**Supplement B**

Table B1: Regression results of the outcome analysis

|  | **Inpatient hospital admissions** | | | **Emergency department admissions** | | | **Standard imaging** | | | **Advanced imaging** | | |
| --- | --- | --- | --- | --- | --- | --- | --- | --- | --- | --- | --- | --- |
| Characteristic | Beta | 95% CI | p-value | Beta | 95% CI | p-value | Beta | 95% CI | p-value | Beta | 95% CI | p-value |
| DiD | -0.09 | -0.33, 0.15 | 0.40 | -0.16* | -0.31, 0.00 | 0.051 | -0.10 | -0.83, 0.63 | 0.80 | -0.16 | -0.80, 0.49 | 0.60 |
| Time | -0.11* | -0.23, 0.01 | 0.064 | -0.03 | -0.10, 0.05 | 0.50 | -0.01 | -0.29, 0.28 | >0.90 | -0.01 | -0.30, 0.28 | >0.90 |
| Group | 0.08 | -0.27, 0.42 | 0.70 | 0.25* | 0.02, 0.48 | 0.032 | 0.11 | -0.84, 1.1 | 0.80 | 0.00 | -0.83, 0.83 | >0.90 |
| Elixhauser Comorbidity Index | 0.10*** | 0.08, 0.12 | <0.001 | 0.05*** | 0.04, 0.07 | <0.001 | 0.11*** | 0.06, 0.15 | <0.001 | 0.08*** | 0.04, 0.13 | <0.001 |
| N | 2,473 |  |  | 2,473 |  |  | 2,473 |  |  | 2,473 |  |  |
| AIC | 6,278 |  |  | 3,886 |  |  | 11,896 |  |  | 11,510 |  |  |
| BIC | 6,307 |  |  | 3,915 |  |  | 11,925 |  |  | 11,539 |  |  |
| Deviance | 3,996 |  |  | 2,655 |  |  | 7,742 |  |  | 8,536 |  |  |

DiD: difference-in-differences estimate; CI: Confidence interval; Poisson-distributed GLM with identity-link, robust standard errors, and adjusted for the pre-trial Elixhauser Comorbidity Index;

*** p<0.01, ** p<0.05, * p<0.1

Note: AIC, BIC are not comparable across the four models due to varying independent variables.

Table B2: Average costs, by group and observation period

| **Parameter** | **t_0_** | | **t_1_** | |
| --- | --- | --- | --- | --- |
|  | **Intervention^1^** | **Control^1^** | **Intervention^1^** | **Control^1^** |
| Total costs | | | | |
|  | 12,846.86 (19,370.70) | 13,492.43 (22,676.81) | 11,134.15 (19,678.37) | 14,171.88 (21,577.33) |
| Healthcare resource utilisation | | | | |
| Total | 12,846.86 (19,370.70) | 13,492.43 (22,676.81) | 11,012.15 (19,678.37) | 14,171.88 (21,577.33) |
| Inpatient Care | 5,841.22 (13,845.65) | 5,243.41 (14,439.12) | 4,601.80 (13,488.27) | 4,569.77 (11,106.09) |
| Outpatient care | 1,125.12 (1,069.09) | 1,235.20 (1,466.28) | 1,089.16 (1,080.56) | 1,420.29 (2,621.83) |
| Pharmaceuticals | 2,212.23 (6,726.36) | 3,462.13 (11,622.95) | 2,558.04 (6,566.21) | 3,811.38 (11,554.18) |
| Other care services | 3,668.29 (7,548.65) | 3,551.69 (8,061.47) | 2,763.15 (6,819.16) | 4,370.44 (10,060.55) |
| Nursing care | 2,138.42 (5,391.48) | 1,985.11 (5,031.21) | 1,567.48 (4,681.44) | 2,292.44 (4,952.12) |
| Therapeutic services | 835.67 (1,742.41) | 789.81 (1,766.53) | 741.65 (1,587.69) | 1,073.37 (2,387.79) |
| Assistive devices | 458.57 (1,201.46) | 639.29 (2,881.46) | 345.96 (1,007.75) | 806.54 (5,350.20) |
| Home nursing care | 235.63 (1,157.06) | 137.48 (846.25) | 108.06 (561.81) | 198.08 (938.92) |
| S2C programme costs |  |  |  |  |
| Total | 0.00 | 0.00 | 122.00 | 0.00 |
| N | 324 | 1,627 | 75 | 447 |

^1^ Mean (SD)


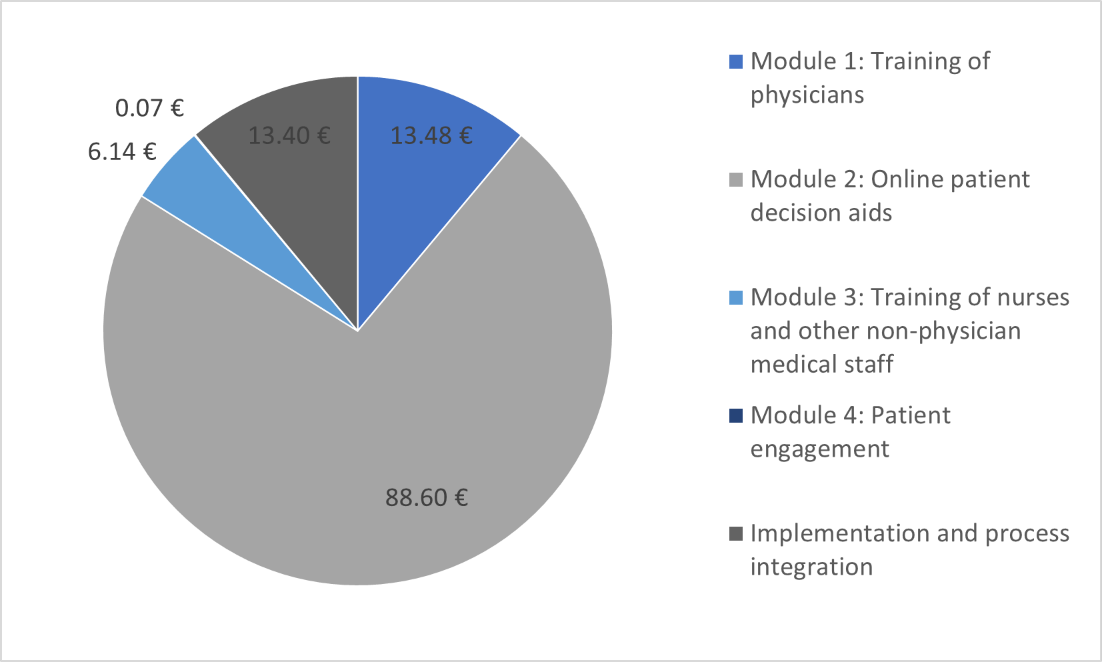


Figure B1: Implementation costs of S2C by module

Table B3: Regression results of the cost analysis

|  | **Total costs** | | | **Total healthcare costs** | | |
| --- | --- | --- | --- | --- | --- | --- |
| Characteristic | Beta | 95% CI | p-value | Beta | 95% CI | p-value |
| DiD | -2,395 | -5,508, 717 | 0.130 | -2,538 | -5,643, 567 | 0.110 |
| Time | -1,030 | -3,176, 1,117 | 0.30 | -1,030 | -3,177, 1,117 | 0.30 |
| Group | 537 | -4,345, 5,420 | 0.80 | 680 | -4,197, 5,556 | 0.80 |
| Elixhauser Comorbidity Index | 2,078*** | 1,698, 2,458 | <0.001 | 2,080*** | 1,700, 2,460 | <0.001 |
| N | 2,473 |  |  | 2,473 |  |  |
| AIC | 49,698 |  |  | 49,626 |  |  |
| BIC | 49,733 |  |  | 49,661 |  |  |
| Deviance | 8,040 |  |  | 8,160 |  |  |

DiD: difference-in-differences estimate; CI: Confidence interval; Gamma-distributed GLM with identity-link, robust standard errors, and adjusted for the pre-trial Elixhauser Comorbidity Index; *** p<0.01, ** p<0.05, * p<0.1

Note: AIC, BIC are not comparable across the two models due to varying independent variables.

Table B4: Sensitivity analysis of outcome parameters: Inpatient hospital admissions

|  | **CCI control,**  **Poisson GLM, log-link^1^** | | | **COVID-19 diagnosis control,**  **Poisson GLM, log-link^1^** | | | **Bias-adjusted cluster-robust standard errors^2^** | | | **Negative binomial GLM,**  **log-link^1^** | | |
| --- | --- | --- | --- | --- | --- | --- | --- | --- | --- | --- | --- | --- |
| Characteristic | IRR | 95% CI | p-value | IRR | 95% CI | p-value | IRR | 95% CI | p-value | IRR | 95% CI | p-value |
| DiD | 0.88 | 0.56, 1.40 | 0.60 | 0.84 | 0.54, 1.32 | 0.40 | 0.91 | 0.56, 1.59 | 0.80 | 0.88 | 0.56, 1.39 | 0.60 |
| Time | 0.85* | 0.71, 1.02 | 0.087 | 0.78** | 0.65, 0.93 | 0.007 | 0.82* | 0.66, 1.01 | 0.066 | 0.83** | 0.69, 0.99 | 0.042 |
| Group | 1.11 | 0.61, 1.99 | 0.70 | 1.15 | 0.65, 2.04 | 0.60 | 1.05 | 0.57, 1.93 | 0.90 | 1.11 | 0.62, 1.98 | 0.70 |
| Elixhauser Comorbidity Index |  |  |  | 1.12*** | 1.09, 1.14 | <0.001 | 1.12*** | 1.10, 1.14 | <0.001 | 1.13*** | 1.10, 1.16 | <0.001 |
| Charlson Comorbidity Index | 1.09*** | 1.07, 1.11 | <0.001 |  |  |  |  |  |  |  |  |  |
| COVID-19 diagnosis |  |  |  | 4.42*** | 2.58, 7.58 | <0.001 |  |  |  |  |  |  |
| N | 2,473 |  |  | 2,473 |  |  | 2,469 |  |  | 2,473 |  |  |
| AIC | 6,373 |  |  | 6,264 |  |  | 6,288 |  |  | 5,675 |  |  |
| BIC | 6,402 |  |  | 6,299 |  |  | 6,317 |  |  | 5,710 |  |  |
| Deviance | 4,090 |  |  | 3,980 |  |  | 4,010 |  |  | 2,106 |  |  |

DiD: difference-in-differences estimate; CI: Confidence interval; IRR: Incidence Rate Ratio; ^1^ robust standard errors; ^2^ clustered at the state of recruitment; *** p<0.01, ** p<0.05, * p<0.1

Note: AIC, BIC are not comparable across the four models due to varying independent variables.

Table B5: Sensitivity analysis of outcome parameters: Emergency department admissions

|  | **CCI control,**  **Poisson GLM, log-link^1^** | | | **COVID-19 diagnosis control,**  **Poisson GLM, log-link^1^** | | | **Bias-adjusted cluster-robust standard errors^2^** | | | **Negative binomial GLM,**  **log-link^1^** | | |
| --- | --- | --- | --- | --- | --- | --- | --- | --- | --- | --- | --- | --- |
| Characteristic | IRR | 95% CI | p-value | IRR | 95% CI | p-value | IRR | 95% CI | p-value | IRR | 95% CI | p-value |
| DiD | 0.65 | 0.37, 1.15 | 0.140 | 0.60* | 0.33, 1.08 | 0.090 | 0.68 | 0.38, 1.23 | 0.20 | 0.62 | 0.35, 1.11 | 0.110 |
| Time | 0.88 | 0.68, 1.14 | 0.30 | 0.77** | 0.60, 0.98 | 0.036 | 0.84 | 0.61, 1.15 | 0.30 | 0.87 | 0.67, 1.14 | 0.30 |
| Group | 2.01* | 0.96, 4.17 | 0.063 | 2.14** | 1.02, 4.51 | 0.045 | 1.88* | 0.92, 3.83 | 0.084 | 2.12* | 1.02, 4.21 | 0.045 |
| Elixhauser Comorbidity Index |  |  |  | 1.14*** | 1.11, 1.17 | <0.001 | 1.14*** | 1.12, 1.16 | <0.001 | 1.16*** | 1.12, 1.19 | <0.001 |
| Charlson Comorbidity Index | 1.11*** | 1.09, 1.14 | <0.001 |  |  |  |  |  |  |  |  |  |
| COVID-19 diagnosis |  |  |  | 6.90*** | 2.73, 17.5 | <0.001 |  |  |  |  |  |  |
| N | 2,473 |  |  | 2,473 |  |  | 2,469 |  |  | 2,473 |  |  |
| AIC | 3,942 |  |  | 3,858 |  |  | 3,888 |  |  | 3,597 |  |  |
| BIC | 3,971 |  |  | 3,893 |  |  | 3,917 |  |  | 3,632 |  |  |
| Deviance | 2,711 |  |  | 2,625 |  |  | 2,659 |  |  | 1,517 |  |  |

DiD: difference-in-differences estimate; CI: Confidence interval; IRR: Incidence Rate Ratio; ^1^ robust standard errors; ^2^ clustered at the state of recruitment; *** p<0.01, ** p<0.05, * p<0.1

Note: AIC, BIC are not comparable across the four models due to varying independent variables.

Table B6: Sensitivity analysis of outcome parameters: Standard imaging

|  | **CCI control,**  **Poisson GLM, log-link^1^** | | | **COVID-19 diagnosis control,**  **Poisson GLM, log-link^1^** | | | **Bias-adjusted cluster-robust standard errors^2^** | | | **Negative binomial GLM,**  **log-link^1^** | | |
| --- | --- | --- | --- | --- | --- | --- | --- | --- | --- | --- | --- | --- |
| Characteristic | IRR | 95% CI | p-value | IRR | 95% CI | p-value | IRR | 95% CI | p-value | IRR | 95% CI | p-value |
| DiD | 0.94 | 0.63, 1.39 | 0.70 | 0.96 | 0.65, 1.43 | 0.90 | 0.94 | 0.73, 1.22 | 0.60 | 0.95 | 0.64, 1.40 | 0.80 |
| Time | 1.01 | 0.87, 1.17 | >0.90 | 1.00 | 0.87, 1.16 | >0.90 | 1.00 | 0.87, 1.14 | >0.90 | 1.00 | 0.86, 1.15 | >0.90 |
| Group | 1.09 | 0.65, 1.82 | 0.70 | 1.05 | 0.63, 1.76 | 0.80 | 1.08 | 0.79, 1.49 | 0.50 | 1.07 | 0.64, 1.77 | 0.80 |
| Elixhauser Comorbidity Index |  |  |  | 1.05*** | 1.03, 1.06 | <0.001 | 1.05*** | 1.03, 1.07 | <0.001 | 1.05*** | 1.03, 1.07 | <0.001 |
| Charlson Comorbidity Index | 1.03*** | 1.01, 1.06 | 0.001 |  |  |  |  |  |  |  |  |  |
| COVID-19 diagnosis |  |  |  | 0.30** | 0.13, 0.66 | 0.003 |  |  |  |  |  |  |
| N | 2,473 |  |  | 2,473 |  |  | 2,469 |  |  | 2,473 |  |  |
| AIC | 11,943 |  |  | 11,897 |  |  | 11,861 |  |  | 9,269 |  |  |
| BIC | 11,968 |  |  | 11,932 |  |  | 11,890 |  |  | 9,304 |  |  |
| Deviance | 7,785 |  |  | 7,740 |  |  | 7,720 |  |  | 2,560 |  |  |

DiD: difference-in-differences estimate; CI: Confidence interval; IRR: Incidence Rate Ratio; ^1^ robust standard errors; ^2^ clustered at the state of recruitment; *** p<0.01, ** p<0.05, * p<0.1

Note: AIC, BIC are not comparable across the four models due to varying independent variables.

Table B7: Sensitivity analysis of outcome parameters: Advanced imaging

|  | **CCI control,**  **Poisson GLM, log-link^1^** | | | **COVID-19 diagnosis control,**  **Poisson GLM, log-link^1^** | | | **Bias-adjusted cluster-robust standard errors^2^** | | | **Negative binomial GLM,**  **log-link^1^** | | |
| --- | --- | --- | --- | --- | --- | --- | --- | --- | --- | --- | --- | --- |
| Characteristic | IRR | 95% CI | p-value | IRR | 95% CI | p-value | IRR | 95% CI | p-value | IRR | 95% CI | p-value |
| DiD | 0.97 | 0.54, 1.74 | >0.90 | 0.92 | 0.51, 1.64 | 0.80 | 0.96 | 0.49, 1.88 | >0.90 | 0.90 | 0.53, 1.57 | 0.70 |
| Time | 0.97 | 0.80, 1.18 | 0.80 | 0.95 | 0.78, 1.15 | 0.60 | 0.97 | 0.81, 1.16 | 0.70 | 0.99 | 0.81, 1.20 | 0.90 |
| Group | 0.93 | 0.46, 1.90 | 0.80 | 0.99 | 0.49, 2.01 | >0.90 | 0.94 | 0.42, 2.07 | 0.90 | 0.99 | 0.50, 1.96 | >0.90 |
| Elixhauser Comorbidity Index |  |  |  | 1.05*** | 1.03, 1.08 | <0.001 | 1.05** | 1.01, 1.10 | 0.025 | 1.06*** | 1.03, 1.09 | <0.001 |
| Charlson Comorbidity Index | 1.07*** | 1.04, 1.10 | <0.001 |  |  |  |  |  |  |  |  |  |
| COVID-19 diagnosis |  |  |  | 2.85*** | 1.89, 4.29 | <0.001 |  |  |  |  |  |  |
| N | 2,473 |  |  | 2,473 |  |  | 2,469 |  |  | 2,473 |  |  |
| AIC | 11,457 |  |  | 11,492 |  |  | 11,498 |  |  | 7,701 |  |  |
| BIC | 11,486 |  |  | 11,527 |  |  | 11,527 |  |  | 7,736 |  |  |
| Deviance | 8,483 |  |  | 8,516 |  |  | 8,529 |  |  | 2,101 |  |  |

DiD: difference-in-differences estimate; CI: Confidence interval; IRR: Incidence Rate Ratio; ^1^ robust standard errors; ^2^ clustered at the state of recruitment; *** p<0.01, ** p<0.05, * p<0.1

Note: AIC, BIC are not comparable across the four models due to varying independent variables.

Table B8: Sensitivity analysis: Total costs

|  | **CCI control,**  **Gamma GLM, log-link^1^** | | | **COVID-19 diagnosis control,**  **Gamma GLM, log-link^1^** | | | **Bias-adjusted cluster-robust standard errors^2^** | | |
| --- | --- | --- | --- | --- | --- | --- | --- | --- | --- |
| Characteristic | Beta | 95% CI | p-value | Beta | 95% CI | p-value | Beta | 95% CI | p-value |
| DiD | -0.16 | -0.58, 0.27 | 0.50 | -0.28 | -0.67, 0.11 | 0.20 | -0.20 | -0.54, 0.13 | 0.20 |
| Time | 0.00 | -0.16, 0.17 | >0.90 | -0.08 | -0.25, 0.09 | 0.40 | -0.05 | -0.26, 0.15 | 0.60 |
| Group | 0.09 | -0.44, 0.63 | 0.70 | 0.17 | -0.34, 0.68 | 0.50 | 0.10 | -0.32, 0.52 | 0.60 |
| Elixhauser Comorbidity Index |  |  |  | 0.14*** | 0.12, 0.16 | <0.001 | 0.14*** | 0.11, 0.17 | <0.001 |
| Charlson Comorbidity Index | 0.13*** | 0.11, 0.15 | <0.001 |  |  |  |  |  |  |
| COVID-19 diagnosis |  |  |  | 1.2*** | 0.73, 1.7 | <0.001 |  |  |  |
| N | 2,473 |  |  | 2,469 |  |  | 2,473 |  |  |
| AIC | 49,746 |  |  | 49,705 |  |  | 49,605 |  |  |
| BIC | 49,781 |  |  | 49,746 |  |  | 49,640 |  |  |
| Deviance | 8,154 |  |  | 8,052 |  |  | 8,053 |  |  |

DiD: difference-in-differences estimate; CI: Confidence interval; ^1^ robust standard errors; ^2^ clustered at the state of recruitment; *** p<0.01, ** p<0.05, * p<0.1

Note: AIC, BIC are not comparable across the four models due to varying independent variables.

Table B9: Sensitivity analysis: Healthcare resource utilisation costs

|  | **CCI control,**  **Gamma GLM, log-link^1^** | | | **COVID-19 diagnosis control,**  **Gamma GLM, log-link^1^** | | | **Bias-adjusted cluster-robust standard errors^2^** | | |
| --- | --- | --- | --- | --- | --- | --- | --- | --- | --- |
| Characteristic | Beta | 95% CI | p-value | Beta | 95% CI | p-value | Beta | 95% CI | p-value |
| DiD | -0.17 | -0.60, 0.26 | 0.40 | -0.30 | -0.69, 0.10 | 0.140 | -0.22 | -0.56, 0.12 | 0.20 |
| Time | 0.00 | -0.16, 0.17 | >0.90 | -0.08 | -0.25, 0.09 | 0.40 | -0.05 | -0.26, 0.15 | 0.60 |
| Group | 0.11 | -0.43, 0.64 | 0.70 | 0.19 | -0.33, 0.70 | 0.50 | 0.11 | -0.31, 0.53 | 0.60 |
| Elixhauser Comorbidity Index |  |  |  | 0.14*** | 0.12, 0.16 | <0.001 | 0.14*** | 0.11, 0.17 | <0.001 |
| Charlson Comorbidity Index | 0.13*** | 0.11, 0.15 | <0.001 |  |  |  |  |  |  |
| COVID-19 diagnosis |  |  |  | 1.2*** | 0.73, 1.7 | <0.001 |  |  |  |
| N | 2,473 |  |  | 2,469 |  |  | 2,473 |  |  |
| AIC | 49,673 |  |  | 49,633 |  |  | 49,533 |  |  |
| BIC | 49,705 |  |  | 49,674 |  |  | 49,568 |  |  |
| Deviance | 8,274 |  |  | 8,172 |  |  | 8,173 |  |  |

DiD: difference-in-differences estimate; CI: Confidence interval; ^1^ robust standard errors; ^2^ clustered at the state of recruitment; *** p<0.01, ** p<0.05, * p<0.1

Note: AIC, BIC are not comparable across the four models due to varying independent variables.
